# Impact of anticancer drugs on the therapeutic efficacy and side effects of hepatic arterial embolization for hepatocellular carcinoma

**DOI:** 10.1002/jgh3.12997

**Published:** 2023-11-10

**Authors:** Hironobu Ihira, Tetsuo Sonomura, Ayano Makitani, Kazuhiro Makitani, Kodai Fukuda, Ryota Tanaka, Takao Koyama, Hirotatsu Sato, Ke Wan, Masaki Ueno, Yoshiyuki Ida, Nobuyuki Kawai, Hiroki Minamiguchi

**Affiliations:** ^1^ Department of Radiology Wakayama Medical University Wakayama Japan; ^2^ Clinical Study Support Center Wakayama Medical University Wakayama Japan; ^3^ Second Department of Surgery Wakayama Medical University Wakayama Japan; ^4^ Second Department of Internal Medicine Wakayama Medical University Wakayama Japan

**Keywords:** gelatin sponge, therapeutic chemoembolization, therapeutic embolization

## Abstract

**Background and Aim:**

Transcatheter arterial chemoembolization (TACE) using various anticancer drugs is often performed to treat hepatocellular carcinoma (HCC). We aimed to compare the therapeutic efficacy and side effects of TACE with anticancer drugs *versus* transcatheter arterial embolization (TAE) without anticancer drugs for HCC.

**Methods:**

Patients with HCC were randomized to either the TACE or TAE group. Up to five target nodules were treated in each patient. Lipiodol (Lp; 10 mL), contrast media (CM; 10 mL), epirubicin (40 mg), mitomycin C (10 mg), miliplatin (70 mg), and 1–2‐mm 2‐day soluble gelatin sponge particles (2D‐SGS) were injected into the TACE group, whereas Lp (10 mL), CM (10 mL), and 2D‐SGS were injected into the TAE group. Treatment effect (TE) of the target nodules was graded (TE1–TE4) and patient responses were assessed. Three months after treatment, blood tests were performed to compare tumor markers and adverse events.

**Results:**

Fifty‐four patients and 161 target nodules were included; 75 nodules in 28 patients were treated by TACE, and 86 nodules in 26 patients were treated by TAE. The number of nodules graded TE1, TE2, TE3, and TE4 was 1, 28, 7, and 39, respectively, in the TACE group and 2, 25, 7, and 52, respectively, in the TAE group. The response rates were 89% (25/28) and 73% (19/26) in the TACE and TAE groups, respectively. There were no significant differences in TE, response rates, or blood test results between the two groups.

**Conclusion:**

In hepatic arterial embolization for HCC, anticancer drugs did not have any impact on the therapeutic efficacy or side effects at 3 months after embolization.

## Graphical abstract

We compared the therapeutic efficacy of transcatheter arterial chemoembolization *versus* transcatheter arterial embolization for hepatocellular carcinoma. The target nodules were classified as TE1–TE4 based on the treatment effect (TE). We found TE was not significantly different between the two groups (*P* = 0.877).

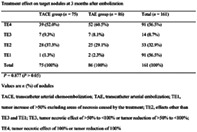

## Introduction

Transcatheter arterial chemoembolization (TACE) using various anticancer drugs is often performed to treat hepatocellular carcinoma (HCC). The antitumor effects of TACE are considered to be achieved by two factors, namely the ischemic effect of occluding the hepatic artery, and the effects of the injected anticancer drugs.^1^ Anticancer drugs such as epirubicin (EP), mitomycin C (MMC), cisplatin, and doxorubicin^2^ have been used to embolize HCC since the clinical introduction of TACE.^1^ It has been reported that TACE for locally advanced HCC increases survival compared with the best supportive care.^3^, ^4^ However, the impact of anticancer drugs on the antitumor effectiveness of TACE and liver damage is not well understood. Chang *et al*. compared the tumor response rate between patients who underwent TACE with cisplatin and those who underwent transcatheter arterial embolization (TAE) without anticancer drugs and found no significant difference between the two groups.^5^ A meta‐analysis in 2002 demonstrated the efficacy of TACE on overall survival, but there was no evidence that it was more effective than TAE, suggesting that the addition of anticancer drugs achieves only a small improvement in therapeutic efficacy.^6^ There are also some reports showing that high doses of anticancer drugs can cause occlusion of the hepatic arteries^7^ or severe liver damage.^8^

The objectives of this study were (i) to compare the therapeutic efficacy and side effects between TACE with anticancer drugs and TAE without anticancer drugs for HCC, and (ii) to elucidate the impact of anticancer drugs on embolization.

## Methods

This clinical study was approved by the Ethical Review Committee of our institution, and informed consent was obtained from all patients.

All procedures performed in studies involving human participants were in accordance with the ethical standards of the Research Ethics Committee of Wakayama Medical University (approval number 1126, date 2012/8/2‐2017/8/1) and with the 1964 Helsinki declaration and its later amendments or comparable ethical standards.

### Patients

The target patients were those with HCC, with up to five target nodules per patient.^9^ In a completely randomized design, patients were assigned to groups of either TACE with anticancer drugs or TAE without anticancer drugs. Randomization was achieved using a random number table (Japanese Industrial Standard Z9031:2001). Patients with hypervascular HCC diagnosed by contrast‐enhanced computed tomography (CT) or magnetic resonance imaging (MRI), Barcelona Clinic Liver Cancer stage A or B, and not on any other treatment were eligible. Patients with any of the following were excluded: tumor invasion into the first branch or trunk of the portal vein (Vp3, Vp4); tumor invasion into the hepatic vein trunk or inferior vena cava (Vv2, Vv3); significant artery–portal vein shunt or artery–hepatic vein shunt; numerous extrahepatic feeding arteries; total bilirubin > 3 mg/dL; estimated glomerular filtration rate < 30 mL/min/1.73 m^2^; massive ascites; or iodine allergy.

### Hepatic arterial embolization

A 4 Fr sheath (Radifocus Introducer IIH, TERUMO, Tokyo, Japan) was inserted into the femoral artery under local anesthesia, and a 4 Fr catheter (RC2 or Shepherd hook, Medikit, Tokyo, Japan) was placed in the celiac and/or superior mesenteric artery. CT during arteriography (CTA; Canon Medical System, Otawara, Japan) and during arterial portography were performed to determine the number, size, and location of liver tumors as well as their invasion into the portal or hepatic veins and their feeding artery. A 1.7 Fr microcatheter (Veloute, ASAHI INTECC, Seto, Japan) was selectively advanced into the feeding artery. For TACE, 10 mL of Lipiodol (Lp; Guerbet, Roissy, France), 10 mL of contrast media (CM), 40 mg of EP (Mylan, Pennsylvania, USA), 10 mg of MMC (Kyowa Kirin, Tokyo, Japan), and 70 mg of miriplatin (Sumitomo Pharma, Tokyo/Osaka, Japan) were mixed to produce 20 mL of Lp‐emulsion. For TAE, 20 mL of Lp‐emulsion (10 mL of Lp and 10 mL of CM) was prepared without the anticancer drugs. The Lp‐emulsion was infused into the feeding arteries, and dense accumulation of Lp in the tumor was confirmed by unenhanced CT. Next, the feeding arteries were embolized with 1–2‐mm 2‐day‐soluble gelatin sponge particles (2D‐SGS), which were prepared from regenerative medicine gelatin (RMG; Jellice, Sendai, Japan). Complete embolization was defined as the complete occlusion of the feeding arteries.

### Post‐treatment assessments

Contrast‐enhanced CT or MRI was performed 3 months after embolization to evaluate the antitumor effect. MRI was used in six patients with a history of iodine allergy. The target nodules were classified as treatment effect (TE)1–4 by three radiologists according to the Response Evaluation Criteria in Cancer of the Liver (RECICL).^10^ Tumor responses were evaluated according to the modified Response Evaluation Criteria in Solid Tumors (mRECIST).^11^ The objective response rate was calculated as (complete response [CR] + partial response [PR])/(CR + PR + stable disease + progressive disease) × 100 (%). Blood tests were performed before and 3 months after embolization to determine albumin, total bilirubin, aspartate aminotransferase (AST), alanine aminotransferase (ALT), γ‐glutamyl transpeptidase, creatinine, estimated glomerular filtration rate, C‐reactive protein, α‐fetoprotein, and protein induced by vitamin K absence‐II (PIVKA‐II). The changes in these values (value after 3 months of embolization − value before embolization) were compared between the two groups.

### Statistical analysis

The proportion of target nodules classified as TE4 after TACE with RMG (143 nodules in 37 patients) was about 50%.^12^ We assumed that the antitumor efficacy of TACE is 50% and that of TAE is 40%. Therefore, a study to verify that TAE is not inferior to TACE within a margin of 10% required treatment of 154 nodules with a one‐sided significance level of 5% and a power of 80%. The TE of the target nodules was evaluated using a mixed‐effects model with treatment as a fixed effect and patients as a random effect. The tumor responses according to mRECIST were compared using Fisher's exact test. Blood test values were compared using *t*‐tests. *P*‐values of <0.05 were considered statistically significant. Statistical analyses were performed using JMP Pro 14.1.0 (SAS Institute Inc., Cary, NC, USA).

## Results

In this study, 59 patients were enrolled between September 2012 and August 2017. Their mean age was 77 ± 8 years (range 53–91 years). There were 43 males and 16 females. Five patients were subsequently excluded: three patients did not undergo CT/MRI evaluation at 3 months after embolization, one patient had evidence of bone metastasis on CT images taken at the time of treatment, and one patient had no viable tumor at the time of embolization. The clinical characteristics of the patients prior to the start of the study are shown in Table 1. A total of 54 patients with 161 target nodes were included in the study; 28 patients with 75 nodes were randomized to TACE, and 26 patients with 86 nodes were randomized to TAE.

**Table 1:** Patient characteristics

Characteristic	TACE group (*n* = 28)	TAE group (*n* = 26)
Age (years)	71 ± 10 (49–90)	77 ± 8 (53–91)
Sex (male/female)	24 (86%)/4 (14%)	16 (62%)/10 (38%)
Child–Pugh (A/B/C)	22 (79%)/6 (21%)/0 (0%)	22 (85%)/4 (15%)/0 (0%)
Hepatitis		
C	16 (57%)	19 (73%)
B	3 (11%)	0 (0%)
Non‐B/non‐C	9 (32%)	7 (27%)
Number of nodules		
1–3	18 (64%)	11 (42%)
≥4	10 (36%)	15 (58%)
Previous treatment		
Surgical hepatectomy	4 (14%)	4 (15%)
PEIT	2 (7%)	0 (0%)
RFA/MCT	13 (46%)	13 (50%)
TACE/TAI	16 (57%)	21 (81%)
RT	1 (4%)	1 (4%)

Values are mean ± SD (range) or *n* (%) of patients.

MCT, microwave coagulation therapy; PEIT, percutaneous ethanol injection therapy; RFA, radiofrequency ablation; RT, radiotherapy; TACE, transcatheter arterial chemoembolization; TAE, transcatheter arterial embolization; TAI, transcatheter arterial infusion.

Table 2 shows the TE of the target nodules. The TE was graded as TE1, TE2, TE3, and TE4 for 1, 28, 7, and 39 nodules, respectively, in the TACE group and for 2, 25, 7, and 52 nodules, respectively, in the TAE group. TE was not significantly different between the two groups (*P* = 0.877).

**Table 2:** Treatment effect on target nodules at 3 months after embolization

	TACE group (*n* = 75)	TAE group (*n* = 86)	Total (*n* = 161)
TE4	39 (52.0%)	52 (60.5%)	91 (56.5%)
TE3	7 (9.3%)	7 (8.1%)	14 (8.7%)
TE2	28 (37.3%)	25 (29.1%)	53 (32.9%)
TE1	1 (1.3%)	2 (2.3%)	91 (56.5%)
Total	75 (100%)	86 (100%)	161 (100%)

*P* = 0.877 (*P* > 0.05).

Values are *n* (%) of nodules.

TACE, transcatheter arterial chemoembolization; TAE, transcatheter arterial embolization; TE1, tumor increase of >50% excluding areas of necrosis caused by the treatment; TE2, effects other than TE3 and TE1; TE3, tumor necrotic effect of >50% to <100% or tumor reduction of >50% to <100%; TE4, tumor necrotic effect of 100% or tumor reduction of 100%.

**Figure 1:**
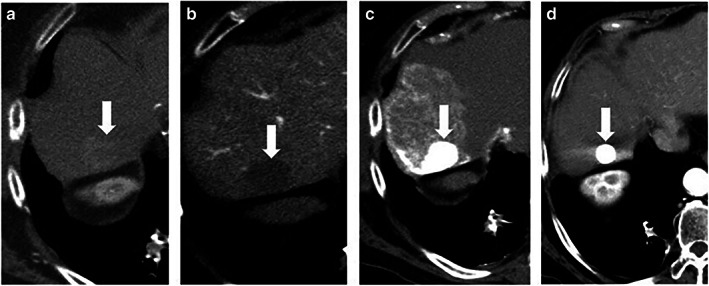
Representative case of a patient with a complete response in the transcatheter arterial chemoembolization (TACE) group. (a) Preoperative computed tomography (CT) during hepatic arteriography shows a tumor (arrow) with early staining in segment 7 of the liver. (b) Preoperative CT during arterial portography shows a defect (arrow) consistent with a tumor. (c) Unenhanced CT performed immediately after TACE shows dense Lipiodol accumulation in the tumor (arrow). (d) Contrast‐enhanced CT performed 3 months after TACE shows shrinkage of the tumor (arrow) without local recurrence.

**Figure 2:**
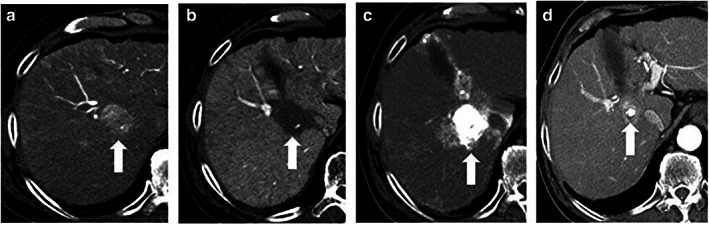
Representative case of a patient with stable disease in the transcatheter arterial chemoembolization (TACE) group. (a) Preoperative computed tomography (CT) during hepatic arteriography showing a tumor (arrow) with early staining in segment 8 of the liver. (b) Preoperative CT during arterial portography shows a defect (arrow) consistent with a tumor. (c) Unenhanced CT immediately after TACE shows dense Lipiodol accumulation in the tumor (arrow). (d) Contrast‐enhanced CT performed 3 months after TACE shows local recurrence (arrow) around the accumulated Lipiodol.

Table 3 shows the tumor responses according to mRECIST. Overall, 25 of 28 patients (89%) in the TACE group and 19 of 26 patients (73%) in the TAE group had good responses (CR or PR); CT images for a representative patient are shown in Figure 1. Meanwhile, 3 of 28 patients (11%) in the TACE group and 7 of 26 patients (27%) in the TAE group had poor responses (stable disease or progressive disease); CT images for a representative patient are shown in Figure 2. The objective response rate was not significantly different between the two groups (*P* = 0.168).

**Table 3:** Tumor responses at 3 months after embolization

	TACE group	TAE group	Total	OR (95% CI)
Effective (CR + PR)	25 (89%)	19 (73%)	44	0.332 (0.049–1.698)
Noneffective (SD + PD)	3 (11%)	7 (26%)	10	
Total	28	26	54	

*P* = 0.168 (*P* >0.05).

Values are *n* (%) of patients.

CI, confidence interval; CR, complete response; OR, odds ratio; PD, progressive disease; PR, partial response; SD, stable disease; TACE, transcatheter arterial chemoembolization; TAE, transcatheter arterial embolization.

Table 4 shows the mean changes in blood test values before and after embolization. There were no significant differences between the two groups.

**Table 4:** Changes in blood test values from baseline to 3 months

	TACE group	TAE group	*P*‐value
Alb (g/dL)	−0.03 ± 0.05	−0.00 ± 0.05	0.671
AST (IU/L)	1.07 ± 5.79	−4.76 ± 5.90	0.483
ALT (IU/L)	2.89 ± 5.76	−2.50 ± 5.87	0.515
γ‐GTP (IU/L)	−11.24 ± 8.98	−11.17 ± 9.36	0.996
T‐bil (mg/dL)	0.08 ± 0.06	0.01 ± 0.06	0.440
Cr (mg/dL)	−0.16 ± 0.15	0.02 ± 0.15	0.371
eGFR (mL/min/1.73 m^2^)	−2.67 ± 2.02	−2.20 ± 2.02	0.873
CRP (mg/dL)	0.09 ± 0.10	0.05 ± 0.11	0.802
AFP (ng/mL)	4200.05 ± 3124.9	350.39 ± 3186.7	0.392
PIVKA‐II (mAu/mL)	4710.2 ± 3714.8	−487.6 ± 3714.8	0.327

Values are mean ± SD.

AFP, α‐fetoprotein; Alb, albumin; ALT, alanine aminotransferase; AST, aspartate aminotransferase; Cr, creatinine; CRP, C‐reactive protein; eGFR, estimated glomerular filtration rate; PIVKA‐II, protein induced by vitamin K absence‐II; T‐bil, total bilirubin; TACE, transcatheter arterial chemoembolization; TAE, transcatheter arterial embolization; γ‐GTP, γ‐glutamyl transpeptidase.

## Discussion

TACE with anticancer drugs is commonly used to treat unresectable HCC. However, the antitumor effect of anticancer drugs is not well understood. A meta‐analysis by Llovet *et al*. concluded that TACE was more effective than TAE in terms of improving the 2‐year survival rate in patients with unresectable HCC.^13^ In addition, a randomized Phase II trial by Meyer *et al*. showed that TACE with cisplatin and polyvinyl alcohol particles achieved a better response rate than TAE with polyvinyl alcohol particles alone.^14^ However, other studies found no significant differences in efficacy between TACE and TAE.^5^, ^6^, ^15^, ^16^ Massarweh *et al*. reported no differences in median survival or risk of death between TACE and TAE in a nationwide retrospective cohort study.^15^ Brown *et al*. reported no significant differences in the response rate, progression‐free survival, or overall survival between TAE with microspheres alone and drug‐eluting bead‐TACE with doxorubicin‐eluting microspheres.^16^ It was also reported that bland TAE is effective for the treatment of giant HCC.^17^ In the present clinical study, the antitumor effect was not significantly different between the TACE and TAE groups. Thus, differences in antitumor effects among studies may be due to the different types and doses of anticancer drugs and embolic materials used.

Sahara *et al*. compared TACE with multiple anticancer drugs (EP, cisplatin, MMC, and 5‐fluorouracil) *versus* TACE with EP alone^7^ and reported no significant differences in the tumor response rates defined according to RECIST^18^ and the European Association for the Study of Liver disease (EASL)^19^ or progression‐free survival between the two groups. However, TACE with multiple anticancer drugs caused significantly more hepatic artery abnormalities and grade 3 AST, ALT elevations, defined according to the Common Terminology Criteria for Adverse Events v4.0 *versus* TACE with EP alone. In an animal study by Nishida *et al*., TACE with high doses of anticancer drugs caused severe liver damage.^8^ In this clinical study, there was no definite evidence of liver damage at 3 months after embolization. However, high doses of multiple anticancer drugs may cause liver damage.

Insoluble gelatin sponges, such as Gelpart (Nippon Kayaku/Astellas, Tokyo, Japan), are often used as embolic materials in TACE for HCC, but the arterial occlusion time of Gelpart is thought to be about 2 weeks.^20^, ^21^ Frequent TACE using insoluble gelatin sponges can cause hepatic artery occlusion, making treatment difficult. To avoid this situation, SGS, which dissolves in about 2 days, was used in this clinical study.^12^, ^22^ TACE with SGS is unlikely to cause hepatic artery occlusion.^22^ In 2007, we developed an RMG with extremely low endotoxicity and antigenicity.^23^ SGS was prepared from RMG by changing the temperature at which the cross‐links are formed and by controlling the dissolution time in saline.^22^, ^24^ Takasaka *et al*. reported that hepatic arteries were completely recanalized at 2 days after embolization using SGS (50 kDa, heat‐induced cross‐linking at 138°C) without the development of collateral pathways in swine.^23^ Kawai *et al*. reported that TACE using 2D‐SGS had similar therapeutic effects and adverse effects to those of TACE using insoluble gelatin sponge particles while causing significantly less hepatic artery impairment.^12^

This clinical study had four limitations. First, the follow‐up period was short (3 months). A longer follow‐up period might reveal significant differences in antitumor efficacy and side effects. Second, adverse events were not evaluated during the acute phase of treatment. The three anticancer drugs used in the TACE group could cause adverse events during the acute phase. Third, the amounts of SGS and anticancer drugs used in each patient are unknown. The endpoints of embolization were dense tumor Lp accumulation, evaluated by unenhanced CT, and the complete occlusion of feeding arteries on angiography. Therefore, the amounts of anticancer drugs and SGS injected varied among the patients. Finally, because this was a single‐center study, a further multicenter randomized study would be desirable.

In conclusion, in hepatic arterial embolization for HCC, anticancer drugs did not impact on the therapeutic efficacy or side effects at 3 months after embolization. TAE without anticancer drugs may be an alternative to TACE with anticancer drugs for patients with poor liver function.

## Informed consent

Informed consent was obtained from all individual participants included in the study.
